# Deep Learning Decoding of Steady-State Visual Evoked Potential (SSVEP) for Real-Time Mobile Brain–Computer Interfaces: A Narrative Review from Laboratory Settings to Lightweight Engineering Applications

**DOI:** 10.3390/brainsci16040387

**Published:** 2026-03-31

**Authors:** Hanzhen Zhang, Chunjing Tao

**Affiliations:** School of Engineering Medicine, Beijing Advanced Innovation Center for Biomedical Engineering, Beihang University, Beijing 100083, China; zhanghanzhen@buaa.edu.cn

**Keywords:** brain-computer interfaces, steady-state visual evoked potential, deep learning, electroencephalography

## Abstract

**Highlights:**

**What are the main findings?**

- This review analyzes deep learning-based steady-state visual evoked potential (SSVEP) decoding methods for mobile real-time BCIs, with a focus on engineering-oriented solutions.
- It highlights three key technological dimensions: lightweight model architectures, ultra-short time window decoding, and cross-subject transfer learning.

**What are the implications of the main findings?**

- The synthesis bridges the gap between algorithmic innovation and practical deployment, providing actionable insights for developing wearable BCI systems.
- The highlighted technologies—lightweight models, short-time window decoding, and cross-subject transfer learning—can accelerate the adoption of SSVEP-BCI in real-world scenarios, such as portable neurorehabilitation devices, hands-free control systems, and vision-assisted wearables.

**Abstract:**

**Background/Objectives**: SSVEP-BCI has broad application potential in mobile human–computer interaction due to its high information transfer rate and stable signal characteristics. The introduction of deep learning technology has significantly advanced SSVEP decoding performance, offering novel approaches for processing short-duration signals and tackling complex classification tasks. The establishment of the Tsinghua Benchmark dataset provides a standardized benchmark for evaluating algorithm performance, accelerating the development of deep learning-based SSVEP decoding. However, a summary of SSVEP deep learning decoding technologies for real-time mobile applications is lacking. **Methods**: We conducted a comprehensive literature review of SSVEP deep learning decoding studies published since 2023, using the Tsinghua Benchmark dataset. This review focuses on technical developments targeting real-time performance, low computational complexity, and high robustness. **Results**: We summarize the key technologies developed for real-time mobile SSVEP decoding. Our analysis thoroughly examines how these techniques address core challenges in the engineering implementation of mobile brain–computer interfaces, including real-time processing requirements, resource constraints, and environmental robustness. **Conclusions**: This review provides a comprehensive overview of SSVEP deep learning decoding technologies for mobile applications, establishing a technical foundation to advance mobile brain–computer interfaces from laboratory settings to practical deployment.

## 1. Introduction

Brain–Computer Interface (BCI) technology, which establishes direct communication pathways between the human brain and external devices, has seen extensive research and application in the fields of neurological rehabilitation, assistive communication, and human–computer interaction [1,2]. Among numerous electroencephalography (EEG) paradigms, steady-state visual evoked potentials (SSVEPs) have emerged as a preferred solution for real-time BCIs due to their high information transfer rate (ITR), extensive target-recognition capabilities, and low user training requirements [3,4]. However, in practical applications, such as mobile scenarios, SSVEP signals are susceptible to background noise and exhibit significant inter-subject variability. Consequently, achieving high-precision, robust signal decoding within short time windows remains the core bottleneck constraining the large-scale engineering application of SSVEP-BCI.

In recent years, deep learning (DL) has achieved breakthrough progress in fields such as computer vision and natural language processing [5,6]. Its powerful nonlinear feature-extraction capabilities and automatic characterization-learning mechanism have prompted researchers to explore the potential of deep learning in EEG signal processing [7,8,9]. Compared with traditional algorithms that rely on manual feature extraction, deep learning can capture complex spatiotemporal and frequency-domain features in EEG signals through endogenous mechanisms and has gradually become the mainstream research direction in SSVEP decoding [10,11,12].

The emergence of standardized datasets has played a pivotal role in advancing algorithmic evolution. The SSVEP Benchmark dataset [13], released by Tsinghua University, has become a globally recognized benchmark for evaluating SSVEP algorithms. With 40 target categories and 35 subject samples, and rigorous experimental protocols, it not only provides a unified testing platform for various decoding algorithms but also offers sufficient training samples for deep learning models. This has significantly facilitated transparent comparisons of algorithmic performance.

Despite the proliferation of related research, existing review work remains limited, with few studies exploring the transition of models from laboratory environments to real-world applications from an engineering perspective focused on “real-time mobile deployment.” This article aims to analyze deep learning-based SSVEP decoding algorithms using the Tsinghua benchmark dataset, which has been available since 2023. It focuses on evaluating the performance and progress of these algorithms in model lightweighting, short-window efficiency improvement, and generalization while also incorporating successful experiences from other fields to provide references for developing high-performance, deployable, real-time mobile brain–computer interface systems.

## 2. Fundamentals of SSVEP Decoding and Evaluation Framework

### 2.1. Tsinghua Benchmark Dataset

**Figure 1:**
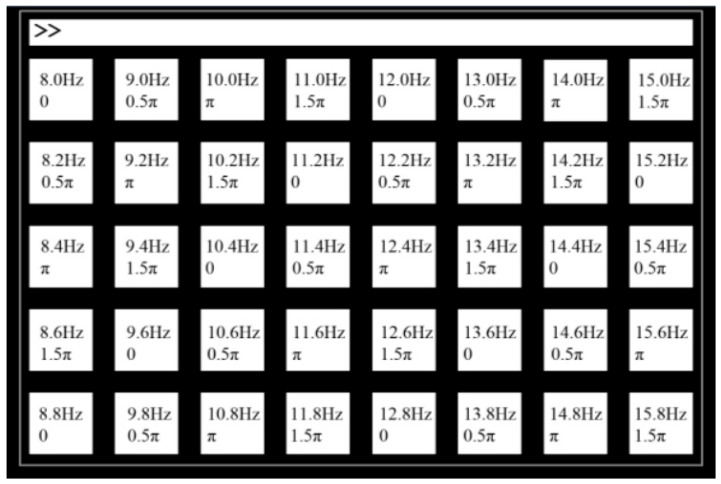
Illustration of Experimental Stimulus Design for Tsinghua Benchmark Dataset Collection [13].

High-quality public datasets are a prerequisite for validating the performance of deep learning algorithms. The SSVEP Benchmark dataset, released by Wang et al. at Tsinghua University, is currently one of the most-cited and authoritative benchmarks in the SSVEP-BCI field [13]. This dataset comprises EEG recordings from 35 subjects using 64-channel wet electrodes. As shown in Figure 1, it features 40 stimulus targets within the 8–15.8 Hz frequency range, spaced at 0.2 Hz intervals with phase intervals of 0.5π, and signals acquired at a sampling rate of 1000 Hz. The high sampling rate in the experimental design ensures that the signals contain rich harmonic components, providing ample raw information for deep learning models to capture multimodal features. The use of wet electrodes and stringent recording-environment requirements guarantees signal quality, delivering relatively clean raw data for preprocessing and subsequent training of deep learning models. This dataset not only provides a unified platform for cross-comparison of algorithm performance but also offers the necessary data support for training deep learning models, given its large scale and multi-target characteristics.

### 2.2. SSVEP Decoding Evaluation Metrics

When evaluating the performance of SSVEP decoding algorithms, information transfer rate (ITR) and classification accuracy are two core and interrelated metrics. Classification accuracy directly measures an algorithm’s ability to correctly identify target stimuli and serves as the foundation for assessing decoding effectiveness [14]. However, for real-time BCIs that pursue high efficiency, response speed should also be incorporated into the evaluation metrics alongside accuracy. ITR comprehensively considers classification accuracy, the number of possible targets, and the average time required to make a selection, thereby reflecting the overall communication efficiency of the system [15]. (*Readers interested in the detailed calculation of ITR may refer to online tools such as: https://bci-lab.hochschule-rhein-waal.de/en/itr.html*, accessed on 31 March 2026).

Research indicates that higher ITR is often associated with shorter data time windows and higher accuracy, yet an inherent trade-off exists among these three factors [16]. Shortening the time window reduces system latency and improves response speed, benefiting real-time interaction, but typically yields insufficient signal features, thereby lowering classification accuracy [17]. Conversely, extending the analysis window accumulates more information, improving decoding stability and accuracy, but introduces significant delays that compromise user experience and system real-time performance [18].

In summary, a core challenge in designing lightweight SSVEP decoding algorithms for mobile applications is to minimize the effective decoding time window while maintaining acceptable accuracy. This is achieved by optimizing the algorithmic architecture and feature-extraction strategies, thereby striking an optimal balance among accuracy, latency, and computational efficiency [16].

### 2.3. Evolution of SSVEP Decoding Methods: From Unsupervised to Supervised Approaches

#### 2.3.1. Review of Traditional Decoding Methods

In the field of SSVEP-BCI, traditional decoding methods dominated before the rise of deep learning. Their core principle involves extracting steady-state responses to specific frequency stimuli using signal processing techniques, shifting from unsupervised to supervised approaches.

The FFT-based frequency-domain analysis method is a classic approach first applied to SSVEP recognition. The intensity threshold method based on the Fast Fourier Transform (FFT), proposed by Cheng et al. in 2002, identifies stimulus frequencies by analyzing the spectral characteristics of EEG signals [19]. However, this method requires parameter optimization, such as channel selection, and its frequency resolution depends directly on the data length. It struggles to obtain accurate spectral representations with short data lengths, limiting its application in high-speed BCI systems.

Since it enables multi-channel signal fusion without requiring training data, Canonical Correlation Analysis (CCA) has gradually become a foundational method in this field, eliminating the need for individual calibration data in practical applications. The CCA method proposed by Lin et al. in 2006 identifies target frequencies by maximizing the correlation between EEG signals and artificial sine-cosine signals [20]. Building upon this, Zhang et al. further proposed the Multivariate Synchronization Index (MSI) method [21]. However, such methods lack individual-specific characteristics and rely solely on frequency information in SSVEP signals, neglecting phase information. Consequently, their performance degrades significantly when encountering a large number of stimulus targets or short signal durations [22].

The emergence of Task-Related Component Analysis (TRCA) represents a shift in SSVEP decoding approaches from unsupervised to supervised methods. In 2018, Nakanishi et al. introduced the TRCA method into the SSVEP-BCI domain. This approach extracts task-related components by maximizing the covariance between trials, utilizing the average waveform from individually calibrated data as a template. It inherently incorporates phase and individual-specific information, partially addressing the limitations of CCA-type methods [23]. While TRCA excels under short time windows, it requires substantial calibration data to compute spatial filters, and the calibration process is time-consuming and labor-intensive. Furthermore, visual fatigue can degrade data quality.

These methods established a solid foundation for SSVEP decoding and provided crucial inspiration for the design of subsequent deep learning model architectures. However, traditional approaches exhibit limitations in achieving high-robustness recognition under short-time-window conditions while reducing dependence on calibration data, thereby spurring the introduction of deep learning techniques in this field. Nevertheless, this does not imply that traditional methods have been completely supplanted within the field. On the contrary, they continue to serve as the cornerstone and are still widely employed in current research. In addition to acting as baselines for comparing new models, traditional methods remain the gold standard in studies focused on hardware performance. For instance, in Zhao et al.’s investigation into peri-auricular electrodes for SSVEP paradigms, the TRCA algorithm was utilized to validate the feasibility of the hardware design [24]. Traditional methods will certainly continue to coexist with emerging deep learning approaches in the field, playing an irreplaceable role.

#### 2.3.2. The Emergence of Deep Learning Methods in the Field of SSVEP Decoding

Breakthroughs in deep learning for computer vision and natural language processing have established a new paradigm for SSVEP decoding. As time-series data with spatiotemporal characteristics, EEG signals share similarities with images and text, yet exhibit significant differences, such as low signal-to-noise ratio and strong non-stationarity. Consequently, researchers have begun exploring the transfer of mature deep learning architectures to the specialized domain of EEG signal processing, adapting models to accommodate the unique properties of EEG signals.

The introduction of Convolutional Neural Networks (CNNs) marked the advent of automated feature extraction in SSVEP decoding. In 2018, Lawhern et al. proposed EEGNet, an exceptionally compact and lightweight network built on Depthwise Separable Convolution. This architecture enables automatic learning of spatiotemporal features, establishing itself as a crucial reference and benchmark for subsequent research [25]. Subsequent studies revealed that feeding raw signals directly into networks was suboptimal. Researchers attempted to bridge the gap between raw signals and deep learning models by feeding complex spectra or Fast Fourier Transform (FFT) results into CNNs, thereby enabling better capture of SSVEP harmonic information [26,27]. Furthermore, drawing inspiration from traditional filter-bank approaches, researchers designed multi-branch CNN architectures to separately extract fundamental-frequency and harmonic-stage features for subsequent fusion. This achieved a deep-learning transformation of the logic underlying widely adopted traditional decoding methods, such as Filter Bank Canonical Correlation Analysis (FBCCA) [26].

Although CNN architectures excel at capturing local features, their limited receptive fields hinder effective detection of long-range temporal dependencies and complex inter-channel nonlinear correlations within EEG sequences. With advancements in large-scale models and the maturation of attention mechanisms, SSVEP decoding has entered the era of “global feature modeling.” In 2023, Chen et al. introduced the SSVEPFormer model, pioneering the application of Transformer architectures in this field. This model uses self-attention mechanisms to dynamically assign weights across time points and channels, thereby capturing long-range dependencies in EEG sequences. It significantly outperformed traditional methods and early CNN models [28] in accuracy within extremely short time windows (0.2 s–0.5 s), resolving the trade-off between high recognition accuracy and short response latency in SSVEP decoding.

As model architectures mature, recent research has shifted focus from purely pursuing accuracy in laboratory settings to enhancing the practicality of algorithms in complex real-world environments. Researchers have widely adopted transfer learning and domain adaptation methods to address inter-subject variability while employing data augmentation techniques such as channel correlation reconstruction and generative adversarial networks [29,30]. Current algorithms are tackling engineering bottlenecks such as “zero calibration” and “short time windows,” aiming to achieve truly “plug-and-play” SSVEP-BCI systems.

## 3. Methodology

### 3.1. Literature Search Strategy

This study aims to identify empirical research on SSVEP deep learning decoding algorithms for mobile BCI applications, validated on the Tsinghua University Benchmark dataset. The search was conducted on 28 February 2026, utilizing the Web of Science Core Collection as the primary database, which covers the Science Citation Index Expanded, Social Sciences Citation Index, Arts & Humanities Citation Index, Conference Proceedings Citation Index, and Emerging Sources Citation Index.

The search employed a subject heading strategy covering the title, abstract, author keywords, and Keywords Plus fields. The search query was constructed as follows: TS = (‘SSVEP*’) AND TS = (‘deep learning*’). The time frame was restricted to 1 January 2023 to 28 February 2026, with a language restriction to English, and the document types included Articles, Proceedings Papers, and Meetings.

### 3.2. Inclusion and Exclusion Criteria

The included literature must satisfy all four criteria. Firstly, the study design must be original empirical research reporting quantitative experimental results; reviews, commentaries, editorials, opinion pieces, and purely theoretical derivations without experimental validation are excluded. Secondly, the dataset requirements stipulate that the Tsinghua University SSVEP Benchmark dataset must be explicitly used for algorithm validation, either as the primary validation dataset or as the core comparison dataset in cross-dataset validation. Third, regarding technical methodology, the core method must be a deep learning architecture, including convolutional neural networks, graph neural networks, Transformers and their variants, transfer learning or domain adaptation frameworks, generative models, etc., and the research objective must be explicitly focused on the SSVEP signal classification task. Fourth, regarding application orientation, the research must clearly demonstrate a technical contribution to mobile BCI scenarios by meeting at least one of the following conditions: model lightweighting, low-calibration, or zero-calibration strategies for short-window decoding.

Exclusion criteria cover six categories. EC1: Non-target signal modalities, where the subject of study is not SSVEP, such as P300, motor imagery, or error-related potentials. EC2: Non-deep learning methods, employing traditional machine learning techniques such as common mode analysis, principal component analysis, or filter bank principal component analysis, without any deep learning components. EC3: Incompatible datasets; failure to use or explicitly mention the Tsinghua Benchmark dataset. EC4: Incompatible language or format; publications not in English, or not of the ‘Article’ or ‘Proceedings’ type, such as pure poster presentations or theses. EC5: Duplicate publication; duplicate reports of the same research; the version with the most complete or latest data is retained.

## 4. Key Technologies for Real-Time Decoding and Mobile Deployment: Lightweight and Low Complexity

Although most deep learning models, including SSVEPFormer, have achieved outstanding decoding accuracy on the Tsinghua Benchmark dataset, significant engineering challenges remain in translating these models into real-world applications. Constrained by limited computational resources, storage capacity, and battery life, mobile devices struggle to run deep neural networks with massive parameter counts directly. Furthermore, mobile applications demand extremely low response latency. Therefore, achieving lightweight, low-complexity models while maintaining high recognition accuracy has become a critical step in advancing SSVEP decoding algorithms from laboratory environments to real-world scenarios.

### 4.1. Compact Architecture Design

**Figure 2:**
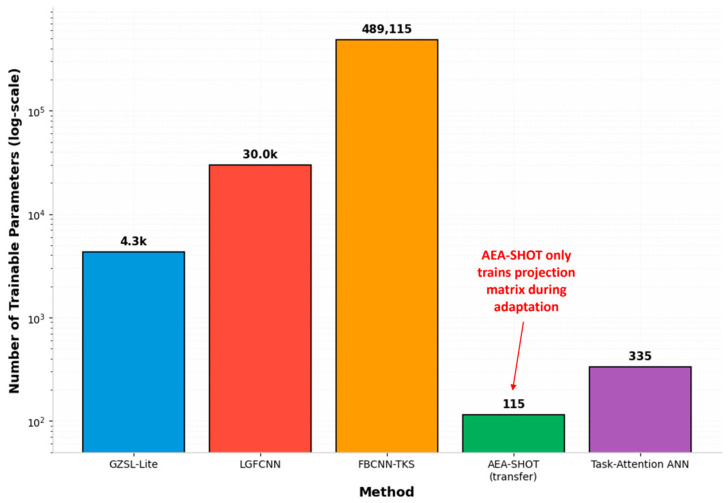
Comparison of trainable parameters between lightweight models.

In the development of mobile BCI systems, constrained by factors such as the size of mobile devices, achieving efficient feature representation with minimal hardware resource overhead remains a core challenge in algorithm design. Addressing this need, recent research has focused on designing compact architectures that integrate EEG physiological features more deeply. The trainable parameters of the models discussed in this chapter, along with the reported performance metrics, are shown in Figure 2 and Table 1, respectively.

Through operator-level weight sharing and optimization of the convolution logic, researchers have achieved a significant reduction in model parameter size. The GZSL-Lite network proposed by Wang et al. [31] employs shared weights to process stimulus features across different frequencies within the feature embedding layer, thereby reducing its parameter count to just 1% of that of existing state-of-the-art (SOTA) methods. Combined with a dynamic sampling start time (DSST) strategy, this network minimizes computational redundancy at its source, achieving a relatively high information transfer rate of 200.28 bits/min (bpm) on the Tsinghua Benchmark dataset, which is roughly 150–170 bpm for general models [31]. Huang et al.’s model [32], combining convolutional neural networks and a selective kernel mechanism (FBCNN-TKS), similarly employs parameter sharing through cross-stimulus convolutional modules and introduces a temporal kernel selection (TKS) mechanism for parallel multi-scale feature extraction. Utilizing lightweight grouped convolutional operators, it achieves an outstanding performance of 251.54 bpm on an extremely short 0.4-s signal length [32]. Similarly, in individual recognition tasks, Wang et al.’s work leverages cross-stimulus weight-sharing strategies [33]. This reduces the number of model parameters by 99.896% compared to standard deep neural networks (DNNs), significantly reducing storage requirements on mobile devices [33].

Transforming traditional signal-processing logic into learnable components within neural networks effectively eliminates model redundancy by leveraging prior knowledge. Ma et al.’s LGFCNN collaborative framework employs lightweight graph-based spatial filters (LGF) to prune redundant information across EEG channels before feature extraction [34]. With only 30.0 k parameters—approximately 42% fewer than the classic lightweight model EEGNet—it substantially alleviates memory constraints on mobile devices [34]. Regarding streamlined attention mechanisms, Wang et al. integrated task attention into compact network design. By mapping spatial filtering operations as constraints on neural weights, their model achieves precise decoding without extensive fully connected layers, demonstrating high recognition efficiency with 0.5-s signal lengths. This physiology-based architectural optimization ensures the model accurately captures SSVEP spatiotemporal features even with minimal parameters [33].

For the engineering vision of “plug-and-play” mobile applications, achieving ultra-low inference latency while maintaining generalization performance has become a critical dimension for evaluating lightweight models. Wang et al. proposed a calibration-free decoding framework that successfully compressed the model size to 0.23 MB—approximately 26.4 times smaller than traditional TFF models—with an inference time of just 10.3 ms, fully meeting real-time interaction requirements [35]. When tackling the complex challenge of cross-subject generalization, Wang’s Adaptive Euclidean Alignment (AEA) method demonstrates extreme lightweight potential: its trainable weights are only 0.013% of a non-transfer learning model, with negligible additional floating-point operations (FLOPs) [36]. By learning only a minimal projection matrix instead of updating the entire network, this approach provides a practical engineering pathway for efficient, real-time generalization processing of large-scale EEG data on computationally constrained mobile devices.

### 4.2. Optimization of Short-Time Window Decoding

**Figure 3:**
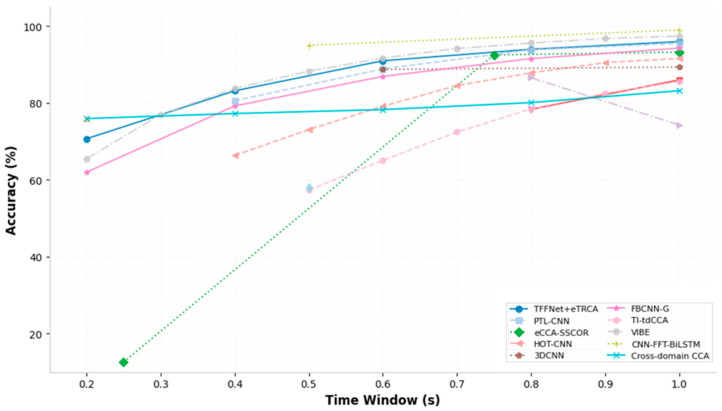
Comparison of Recognition Accuracy for Short Window Models Under Different Window Length Conditions.

**Figure 4:**
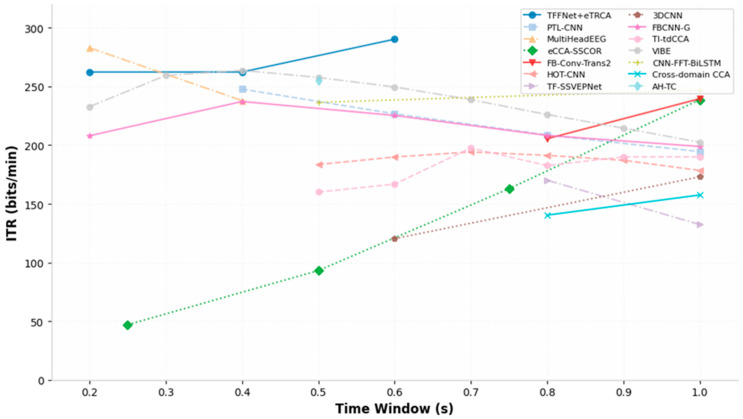
Comparison of ITR Recognition Performance Across Different Window Lengths in the Short Window Model.

In mobile BCI systems, reducing the time window length (TWL) for individual targets is one of the most direct methods to enhance ITR. However, extremely short time windows result in insufficient energy in the dominant frequency component of SSVEP signals in the frequency domain, making them susceptible to being drowned out by broadband noise from background spontaneous EEG. To address this challenge, researchers have proposed techniques such as forward signal prediction, cross-domain data augmentation, extraction of extreme multi-dimensional spatio-temporal-frequency features, and a hybrid decoding and adaptive fusion strategy. The accuracy and ITR under short-window conditions are shown in Figure 3 and Figure 4, which were plotted using data reported in previous related work.

#### 4.2.1. Forward Signal Prediction and Cross-Domain Data Augmentation

To address the inherent limitations of the physical length of short-window signals, researchers have proposed algorithmic approaches to generate additional information. Shen et al.’s spatio-temporal frequency fusion network (TFFNet) employs multi-scale deep separable convolutions (MDSC) to capture transient features, achieving the first accurate prediction of future SSVEP values [37]. By fusing the generated extended signals with the original short data, the framework combining TFFNet and eTRCA (FSE-eTRCA) maintains high recognition accuracy even within an extremely short 0.2-s window. Furthermore, to address the issue of insufficient model training with limited data, researchers introduced data augmentation techniques. Hu et al. explored leveraging transfer learning and domain adaptation (DA) techniques to transfer the global feature distribution from long-duration signals to short-duration inference tasks. This approach endows the model with stronger generalization capabilities without increasing actual fixation duration [38]. This performance enhancement strategy, centered on predictive data, fundamentally alleviates the issue of insufficient information entropy in short windows.

#### 4.2.2. Extraction of Extreme Multi-Dimensional Spatio-Temporal-Frequency Features

In extremely short time slices, traditional one-dimensional feature extraction often loses critical information about phase evolution. Therefore, multi-dimensional feature fusion has become a key to ensuring accuracy. The subject-specific CNN model with parameter-based transfer learning (PTL-CNN), proposed by Ji et al., simultaneously extracts temporal fluctuations and spatial topological relationships of signals through spatio-temporal task-related component analysis (ST-TRCA), thereby significantly improving the signal-to-noise ratio within a 0.3-s window [39]. Oikonomou et al.’s MSSENet employs multi-scale spatio-spectral feature extraction operators to capture harmonic components of varying orders within short durations [40]. Similarly, Chen et al. use the MS-SFNet architecture, which enhances model robustness to frequency drift by processing responses across different frequency bands in parallel branches [41]. Hamou et al. introduced the Dual-Channel Convolutional Residual Network (DCRN), which preserves high-frequency fine features in extremely short signals through residual connections [42].

To address the non-stationarity of short-duration signals, some studies introduced more complex representations. Xia et al. demonstrated that complex-valued convolutional neural networks (Complex-valued CNN) better handle the phase-locked characteristics of SSVEPs, enabling stable phase component extraction even from waveforms shorter than a full cycle [43]. Xiong et al.‘s DCN-SSVEP incorporates deformable convolution, enabling adaptive adjustment of receptive fields based on subjects’ instantaneous EEG rhythms to achieve precise anchoring within 0.4-s windows [44]. Meanwhile, Yang et al. effectively mitigated the vanishing-gradient problem in deep networks when processing short, transient signals by using a residual learning architecture (Res-SSVEPNet), ensuring classification upper bounds even with extremely short windows [45].

#### 4.2.3. Hybrid Decoding Framework and Adaptive Fusion Strategy

Faced with complex noise interference in mobile environments, single algorithms often lack sufficient robustness in short windows, prompting researchers to shift toward adaptive hybrid decoding schemes. Khabbaz et al.’s Adaptive Hybrid Algorithm (AH-TC) dynamically integrates TRCA with Correlation Component Analysis (CORRCA), enabling real-time perception of changes in the signal environment and the allocation of weights [46]. Wen et al.’s FB-TRCA-Net employs filter-bank integration to more deeply couple deep learning with statistical spatial filtering, significantly enhancing classification consistency under ultra-short response times [47]. Additionally, attention mechanisms and global dependency modeling play crucial roles in this process. Niu et al. combined the strengths of convolutional and Transformer architectures (Conv-Transformer), utilizing self-attention mechanisms to weight key sampling points along the time axis [48].

## 5. Cross-Subject Transfer and Low-Calibration Strategy for Plug-And-Play Applications

**Figure 5:**
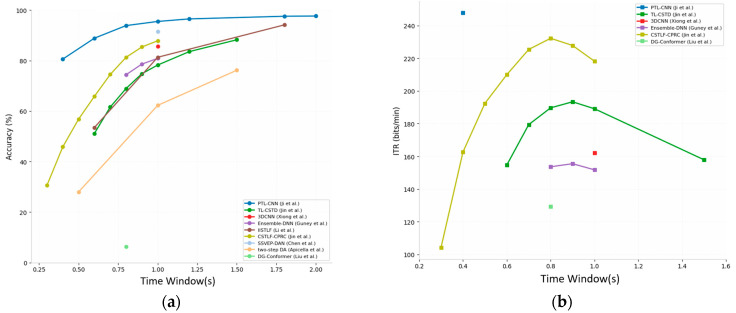
Cross-Subject Model Performance Comparison: (**a**) Comparison of Model Recognition Accuracy Across Different Time Windows; (**b**) Comparison of Model Recognition ITR Across Different Time Windows.

**Figure 6:**
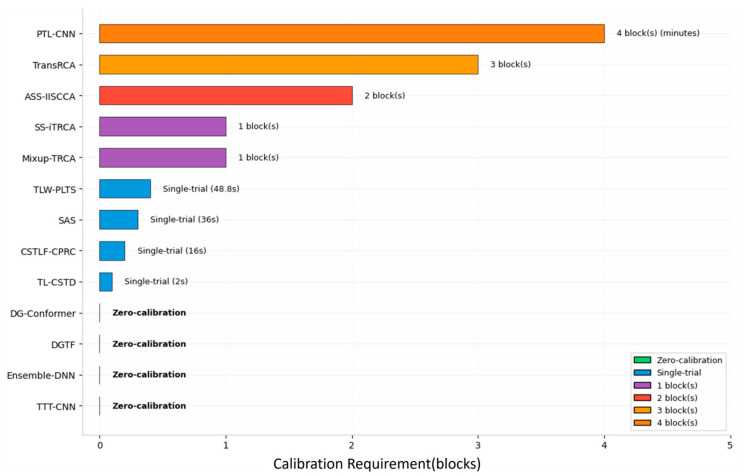
The amount of calibration data required for the model.

Due to variations in skull thickness, electrode placement deviations, and individual differences in cortical folding, SSVEP exhibits significant covariate shifts across subjects and even within the same subject at different time points. This leads to a sharp decline in deep learning model performance across subjects, forcing systems to adopt lengthy individual calibration to maintain recognition accuracy. To overcome this bottleneck, current research paradigms focus on developing “plug-and-play” systems that can be activated with minimal or no training data via transfer learning and generative techniques. The comparison between the model’s basic performance and the required amount of calibration data discussed in this chapter is shown in Figure 5 and Figure 6.

### 5.1. Source-Target Knowledge Mapping and Individualized Instance Transfer

One strategy for achieving low-calibration transfer learning involves establishing a knowledge bridge between the source domain—data collected from a large-scale subject cohort—and the target domain of new subjects. Building on this concept, the Parametric Transfer Learning (PTL) framework proposed by Ji et al. [39] establishes “common ground” across multi-subject datasets. It performs fine-tuning for new users, enabling robust decoding in minimal time [39]. The effectiveness of this “template and spatial filter” joint transfer approach was further validated by Zhang et al. [49], demonstrating superior robustness compared to training from scratch, particularly when training samples are extremely scarce.

To further squeeze calibration efficiency, researchers began exploring the adaptation limits under minimal samples. Jin et al.’s TL-CSTD algorithm demonstrated that, even with only 2 s of single-trial data for calibration, approximation-correction techniques could reduce adaptation time to 4.2% of that of traditional methods [50]. Addressing task-to-task correlations in multi-target systems, Jin et al. also proposed the Cross-Stimulus Transfer Learning Framework (CSTLF-CPRC), which enables knowledge flow between instruction sets by leveraging shared periodic, repetitive components [51]. At the knowledge filtering level, Wang et al.’s similarity-aware subject selection strategy (SS-iTRCA) introduced a dynamic filtering mechanism. By eliminating source instances mismatched with target subject features, it ensures transfer accuracy [52]. Furthermore, Wei et al. and Li et al. explored the possibility of maintaining high ITR with minimal calibration effort, respectively, through CCA framework refinement and intra-/inter-subject knowledge co-transfer approaches [53,54].

### 5.2. Domain-Invariant Representation and Unsupervised Distribution Alignment

In the pursuit of zero calibration, a key research focus is learning feature distributions independent of individual subjects, without access to target-domain labels. Building on domain generalization theory, Huang et al. proposed a preprocessing paradigm for cross-subject BCI that requires no target-domain data by learning domain-invariant spatial filters [55]. This paradigm was further enhanced at the architectural level in the DG-Conformer model proposed by Liu et al. [56], which leverages the global attention mechanism of Transformers, combined with StableNet’s sample weighting technique, to significantly improve the model’s upper bound on generalization to unseen subjects.

To achieve direct feature-space alignment, Chen et al.’s SSVEP-DAN framework narrowed distributional gaps using cross-domain data alignment techniques [57]. Apicella et al. systematically evaluated multiple domain adaptation (DA) methods to select optimal alignment strategies for real-time systems [58]. For more challenging privacy-preserving and offline deployment scenarios, Guney et al. validated a training-free approach based on deep neural network ensembles [59]. To address dynamic adaptation during inference, Duan et al. introduced test-time training, enabling models to adapt to unlabeled samples from current inputs, thereby compensating for feature shifts across subjects in real time [60].

### 5.3. Physiologically Inspired Data Augmentation and Virtual Feature Completion

When real individual calibration data is insufficient to support parameter convergence in deep learning models, researchers leverage the physical properties of EEG signals for “feature reconstruction” to ensure performance. The temporally local weighting-based phase-locked time-shift data augmentation method (TLW-PLTS) proposed by Huang et al. can generate a large number of physiologically consistent augmented samples with minimal raw data support, addressing overfitting at the data level [61].

In deep feature dimension exploration, Xiong et al. investigated deep transfer logic for frequency-domain features [44]. At the same time, Ding et al. enhanced the noise resilience of subject-independent decoders using spatio-temporal energy representation techniques [62]. To address signal distortion in complex environments, Huang et al. successively developed generation methods for shifted and interleaved signals [63] and an augmentation strategy based on the Mixup mechanism [64], which broadens the model’s decision boundaries by simulating real-world noise distributions and phase drifts. Furthermore, Zhang et al. [65] advanced the work by integrating data augmentation with language models, achieving cross-domain fusion from “pure waveform classification” to “semantic logic-assisted” applications in spellers. This provides broader engineering perspectives for high-performance, low-calibration BCI systems.

## 6. Application-Oriented Challenges and Future Outlook: From Laboratory to Engineering Implementation

### 6.1. Performance Validation in Complex Scenarios: Real-World Testing Beyond Benchmark Datasets

Deep learning decoders have demonstrated superior performance in controlled laboratory settings, but their robustness and generalization capabilities in real-world, complex scenarios remain the primary challenge for engineering implementation. Real environments introduce multiple sources of interference and dynamic variations not present in benchmark datasets.

Take the autonomous driving field as an example: testing must cover interaction scenarios with vulnerable road users (VRUs) in complex, high-risk conditions. These scenarios often exhibit long-tail, rare yet critical characteristics [51]. Traditional scenario-based testing methods struggle to systematically explore these high-risk domains, potentially underestimating inherent risks in complex interactions. To generate realistic and diverse boundary scenarios, the Binary Adaptive Deep Reinforcement Learning (BADRL) framework has been proposed. This approach trains AI-driven background agents to evaluate overall vehicle performance while incorporating a scenario complexity model for real-time assessment, dynamically scaling complexity [52].

For SSVEP-BCI systems, current algorithmic research predominantly relies on offline testing using standard datasets collected in laboratory settings, lacking online feasibility and robustness testing. However, for practical applications, SSVEP decoders must undergo rigorous validation in real-world scenarios beyond laboratory resting-state conditions—environments filled with dynamic visual distractions, fluctuating user attention, and nonstationary EEG signals—to ensure reliability and safety.

### 6.2. System Integration and Closed-Loop Interaction: The Role of Deep Learning Decoders in Complete BCI Systems

Integrating high-performance deep learning decoders into a complete, usable BCI system is a critical step in realizing their engineering value. This requires the decoder to function not merely as an isolated signal processing module, but as part of an efficient closed-loop interactive system encompassing stimulus presentation, system control, and user feedback. Such closed-loop systems enable real-time, adaptive adjustments based on neural feedback. For instance, in the field of neuromodulation, the Brain-Machine Interface Neuromodulation Research Tool (BMINT) integrates neurophysiological signal sensing, mainstream machine-learning algorithm computation, and real-time pulse-level electrical stimulation delivery, providing a platform for achieving real-time closed-loop neuromodulation based on edge AI computing [53]. Similarly, the Closed-Loop System for Human Intracranial Stimulation (CLoSES) enables real-time neural feature computation and stimulus triggering via decision algorithms, providing a flexible platform for investigating interactions between brain states and stimulation responses [54]. In robotics, the Integrated Intelligent Tactile System (IITS) functions as a closed-loop system that integrates multi-channel tactile-sensing electronic skin, data acquisition and information processing chips, and feedback control. This enables robots to achieve human-like tactile perception and perform flexible object grasping [55].

For SSVEP-BCIs, deep learning decoders must output stable, accurate commands with ultra-low latency. These commands directly drive external devices or modify stimulus parameters, forming a complete “perception-decoding-control-feedback” closed loop. Such integration imposes stringent engineering demands on system real-time performance, stability, and power consumption.

### 6.3. Standardization, Open-Source Initiatives, and Reproducibility Drive the Advancement of Engineering Processes

Standardized protocols, open-source tools, and reproducibility are cornerstones for accelerating the transition of SSVEP-BCI technology from laboratory prototypes to widespread engineering applications and clinical practice. The absence of unified standards hinders the comparability of the results across research teams and the integration of algorithms, severely impeding technological iteration and industrial collaboration. In magnetic resonance imaging, the adoption of the open Pulseq sequence standard to define chemical exchange saturation transfer (CEST) experiments, coupled with protocol sharing through open-source databases, has effectively promoted multi-center, multi-device compatibility and result reproducibility [56]. In computational biology, adhering to FAIR (Findability, Accessibility, Interoperability, Reusability) principles and leveraging open-source software, widely adopted standards, and public repositories ensures the long-term reproducibility and reusability of computational models, extending their utility beyond the lifecycle of specific software [57]. For deep learning decoding models, this necessitates public disclosure of detailed architecture, training code, hyperparameter settings, and preprocessing workflows. Open-source frameworks such as **MNE-Python** provide a unified Python-based analysis environment for EEG and MEG data, integrating the entire workflow from preprocessing to machine learning analysis. Such frameworks support containerized workflows to achieve fully reproducible analysis pipelines, which is essential for advancing open science practices in BCI researchTherefore, establishing more large-scale benchmark datasets, standardized data formats, and open-source one-stop processing platforms in the SSVEP-BCI field will significantly lower engineering barriers, promote community collaboration, and ensure the reliability and transferability of research outcomes.

### 6.4. Emerging Directions: Integration with Other EEG Paradigms, Adaptive Stimulus Optimization, Etc.

Future SSVEP-BCI systems for engineering applications will no longer be confined to a single steady-state visual evoked potential paradigm. Their development will evolve along emerging directions such as integration with other EEG paradigms and system adaptive optimization. Multimodal fusion enhances the system’s information transfer rate, robustness, and user experience. Integrating SSVEP with paradigms such as motor imagery, P300, or EEG microstates leverages the complementary strengths of different modalities to achieve more natural and efficient hybrid BCI control. However, such multimodal fusion remains technically challenging due to inherent differences in spatial patterns, data distributions, and temporal dynamics across modalities. Balancing the effective use of spatiotemporal coupling characteristics while preserving the distinct properties of each modality is a key challenge that requires further investigation. At the model level, this requires deep learning architectures capable of effectively fusing diverse neural signal features with distinct spatio-temporal characteristics.

On the other hand, adaptive optimization significantly enhances system practicality in real-world applications. On the stimulus side, adaptive stimulus optimization dynamically adjusts frequency, phase, contrast, or spatial location based on the user’s real-time attentional state, fatigue level, or EEG response quality to maintain optimal evoked response. On the decoding side, employing online learning algorithms enables decoding models to adapt to intra- and inter-individual variability, as well as potential neural signal drift over time. SSVEP-BCI systems can establish a joint optimization closed-loop of “stimulus-response-decoding,” utilizing deep learning models to optimize both stimulus parameters and decoding strategies simultaneously. This achieves dynamic maximization of overall system performance, better adapting to complex and dynamic real-world usage environments.

## 7. Discussion

### 7.1. Main Contributions

This paper reviews SSVEP deep learning-based decoding technologies using the Tsinghua Benchmark dataset since 2023, focusing on key engineering challenges for real-time mobile applications. Compared with existing reviews, the main contributions of this paper are as follows:

Firstly, this paper is the first to analyze SSVEP deep learning decoding technologies from an engineering perspective with a narrative method. While existing reviews tend to emphasize algorithmic classification or offline accuracy comparisons, this paper adopts ‘real-time performance, lightweight design and robustness’ as core evaluation dimensions, summarizing three major technical directions: lightweight model architectures, ultra-short-window decoding, and cross-subject transfer learning. This engineering-oriented analytical framework effectively bridges the gap between algorithmic research and practical deployment.

Concurrently, conducting standardized comparisons using an authoritative benchmark dataset is another key feature of this paper. All research cited in this paper is validated using the Tsinghua Benchmark dataset, which features 40 target categories, 35 subjects, and standardized experimental protocols and signal acquisition parameters. This selection ensures comparability between different algorithms, providing readers with a relatively objective performance benchmark.

Furthermore, this paper generally identifies the key technical bottlenecks in the transition from laboratory settings to mobile applications, and introduces some future research directions that can be referenced from other fields. This paper not only summarizes the strengths of existing technologies but also conducts an in-depth analysis of the engineering challenges in real-time systems, including computational resource constraints, individual variations, and environmental noise interference, thereby clarifying key areas for future research.

### 7.2. Limitations of This Study

Although this paper endeavors to provide a comprehensive overview of the latest developments in SSVEP deep learning decoding, the following limitations should be borne in mind when interpreting the results.

Admittedly, whilst using the Tsinghua Benchmark dataset as a unified benchmark for algorithm comparison ensures the objectivity and reproducibility of comparisons, this choice may exclude some potentially beneficial algorithms developed on other datasets or self-collected data. In particular, for mobile BCI systems, the data distribution in real-world application scenarios differs significantly from the benchmark data collected under laboratory conditions. Consequently, the generalization capabilities of the algorithms reviewed in this paper in real-world mobile scenarios remain to be further validated.

Furthermore, the availability of performance data has, to some extent, limited the depth of quantitative comparisons. The field of SSVEP deep learning is still in a phase of rapid development, and no unified standards have yet been established for experimental setups, evaluation metrics, or data partitioning methods across studies. Although this paper attempts to summarize and compare the accuracy and ITR of various algorithms, some studies have not reported complete time-window performance curves or cross-subject generalization results, making it difficult to conduct a more in-depth quantitative comparative analysis. This limitation is due to the field’s current stage of development and underscores the urgency of establishing unified evaluation standards.

It is particularly worth noting that the risk of overfitting and its impact on practical usability remain uncertain. Although the performance metrics reported in various studies are encouraging, the sample size of the Tsinghua Benchmark dataset remains relatively limited for typical deep learning training scales. Existing studies predominantly employ strategies such as data augmentation, transfer learning, and parameter sharing to mitigate overfitting; however, the long-term stability of the models in real-world mobile environments, their cross-scenario generalization capabilities, and the consistency of real-time responses have not yet been fully validated.

In addition, this paper focuses primarily on the models’ performance under typical conditions, with little systematic exploration of scenarios in which the algorithms fail. For instance, the performance limits of existing models remain unclear when users are highly fatigued, when ambient light interference is strong, when electrode contact is poor, or when stimulation parameters are poorly configured. These failure scenarios are precisely the most common challenges in mobile applications, and an in-depth analysis of them would help drive the development of algorithms towards greater robustness and reliability. As this paper does not address these issues specifically, it highlights a direction worthy of attention for future research.

Finally, this review is primarily centered on algorithmic approaches—an emphasis that reflects the focus of the majority of current research. However, in framing this review, it is equally important not to overlook the gaps in hardware optimization and related areas, nor to leave them entirely unaddressed. A more holistic consideration of these aspects would provide a valuable complement to the algorithmic perspective and help chart a more comprehensive roadmap for future development in the field.

## 8. Conclusions

Deep learning technologies have brought about a paradigm shift in brain–computer interfaces based on steady-state visual evoked potentials. Their core contribution lies in significantly enhancing information transfer rates within short time windows through strategies such as end-to-end learning, model lightweighting, and transfer learning while effectively addressing traditional challenges like calibration burdens, individual variability, and noise interference. This shift signifies a transition in research focus from a pure offline accuracy race toward a systematic engineering approach that seeks a dynamic equilibrium between performance, efficiency, universality, and usability.

From a historical perspective, recent research has preliminarily reconciled the tension between high accuracy and low computational overhead through architectural innovations, while transfer learning partially mitigates model dependency on new users, enhancing generalizability. However, this equilibrium remains fragile and preliminary. Complex models pursuing extreme performance often sacrifice computational efficiency and real-time capability, whereas overly lightweight models may exhibit insufficient long-term stability and cross-scenario robustness. Furthermore, most research remains focused on optimizing decoding algorithms themselves, with insufficient synergistic optimization of hardware power constraints, fluctuations in dry-electrode signal quality, and the design of more natural, low-cognitive-load interfaces.

Therefore, future breakthroughs lie not in further improving individual metrics but in constructing an interdisciplinary, collaborative design framework that integrates signal processing, machine learning, low-power hardware, and human factors engineering. Research must evolve from open-loop “decoding-output” models to intelligent closed-loop interaction systems that adapt to user states and environments. Only then can ultimate bottlenecks—long-term stability, ultra-low-power deployment, and seamless user experience—be overcome, propelling SSVEP-BCI from high-performance laboratory prototypes to truly wearable, mass-market practical interaction tools.

## Figures and Tables

**Table 1:** Performance Comparison of Lightweight Design Models.

Method	Max ITR (bits/min)	Best Acc (%)	Parameters	Time of Inference	Key Technique
GZSL-Lite	200.28 (0.6 s)	85.0 (0.5 s)	4270	0.47 ms/trial	Dual-attention DSST strategy
LGFCNN	221.4 (0.6 s)	93.1 ± 4.3 (1.0 s)	30,000	2.1 ms/trial	Graph-based filter 4 lightweight blocks
Fast SSVEP	N/A	35.4 ± 15.6 (0.4 s)	N/A	10.3 ms/trial (0.3 s)	Adaptive Spectrum Denoise
FBCNN-TKS	251.54 (0.4 s)	83.10 ± 2.8 (0.4 s)	489,115	5 ms/subject	Selective Kernel Dilated & Group Conv
AEA-SHOT	N/A	68.0 ± 18.9 (SSVEP)	115	N/A	Adaptive Euclidean Alignment
Task-Attention ANN	182.05 (0.5 s)	85.488 (0.5 s)	335	0.668 s/block	Task-specific Attention

## Data Availability

No new data were created or analyzed in this study. Data sharing is not applicable to this article.

## Abbreviations

The following abbreviations are used in this manuscript:

SSVEP	Steady-State Visual Evoked Potential
BCI	Brain–Computer Interface
EEG	Electroencephalogram/Electroencephalography
DL	Deep Learning
ITR	Information Transfer Rate
mBCI	Mobile Brain–Computer Interface
FFT	Fast Fourier Transform
CCA	Canonical Correlation Analysis
MSI	Multivariate Synchronization Index
TRCA	Task-Related Component Analysis
CNN	Convolutional Neural Network
SOTA	state-of-the-art
DNN	Deep Neural Network
FLOPs	Floating Point Operations
TKS	Time Kernel Selection
AEA	Adaptive Euclidean Alignment
TWL	Time Window Length
MDSC	Multi-scale Depthwise Separable Convolution
ST-TRCA	Spatio-Temporal Task-Related Component Analysis
DA	Domain Adaptation
CORRCA	Correlation Component Analysis
PTL	Parameterized Transfer Learning
CSTLF-CPRC	Cross-Stimulus Transfer Learning Framework using Common Period Repetition Components
SS-iTRCA	Similarity-aware Subject selection for individual TRCA
DG-Conformer	Domain Generalization Conformer
TLW-PLTS	Temporally Local Weighting-based Phase-Locked Time-Shift
VRUs	Vulnerable Road Users
BADRL	Bi-level Adaptive Deep Reinforcement Learning
BMINT	Brain-Machine Interactive Neuromodulation Research Tool
CLoSES	Closed-Loop intracranial Stimulation system
IITS	Integrated Intelligent Tactile System
CEST	Chemical Exchange Saturation Transfer
FAIR	Findable, Accessible, Interoperable, Reusable
GAN	Generative Adversarial Network
PRISMA	Preferred Reporting Items for Systematic Reviews and Meta-Analyses
FMA	Fugl-Meyer Assessment
SVM	Support Vector Machine
